# Supplementary material for: How Does Procedural Justice Affect Job Crafting? The Role of Organizational Psychological Ownership and High-Performance Work Systems
Source: Behav Sci (Basel). 2024 Dec 24;15(1):4. doi: 10.3390/bs15010004 (PMC11763208; doi:10.3390/bs15010004)
Supplement: Supplementary file 1 [file behavsci-15-00004-s001.zip › behavsci-3326546-supplementary.pdf]

Certainly! Below is the English translation of the questionnaire:

For Employees:

1. I will introduce new work methods to improve work processes.

Rating Scale: 1 (Strongly Disagree) to 5 (Strongly Agree)
1 2 3 4 5

2. I will make minor adjustments to work processes to increase efficiency.

Rating Scale: 1 (Strongly Disagree) to 5 (Strongly Agree)
1 2 3 4 5

3. I will adjust my work methods to make the job easier.

Rating Scale: 1 (Strongly Disagree) to 5 (Strongly Agree)
1 2 3 4 5

4. I will rearrange the tools and materials related to my work to make it more convenient.

Rating Scale: 1 (Strongly Disagree) to 5 (Strongly Agree)
1 2 3 4 5

5. I can express my viewpoints in the team (department) decision-making process.

Rating Scale: 1 (Strongly Disagree) to 5 (Strongly Agree)
1 2 3 4 5

6. I can influence the outcome of decisions that affect me.

Rating Scale: 1 (Strongly Disagree) to 5 (Strongly Agree)
1 2 3 4 5

7. Our team (department) maintains consistency in executing the decision-making process.

Rating Scale: 1 (Strongly Disagree) to 5 (Strongly Agree)
1 2 3 4 5

8. Our team (department) is unbiased in the decision-making process.

Rating Scale: 1 (Strongly Disagree) to 5 (Strongly Agree)
1 2 3 4 5

9. The decision-making process of our team (department) is based on accurate and objective judgments.

****Rating Scale**: 1 (Strongly Disagree) to 5 (Strongly Agree)**

1 2 3 4 5

10. Members of our team (department) can suggest improvements for decisions they disagree with.

****Rating Scale**: 1 (Strongly Disagree) to 5 (Strongly Agree)**

1 2 3 4 5

11. Our team (department)'s decision-making process complies with ethical and moral rules.

****Rating Scale**: 1 (Strongly Disagree) to 5 (Strongly Agree)**

1 2 3 4 5

12. I have a strong sense of ownership of the company.

****Rating Scale**: 1 (Strongly Disagree) to 5 (Strongly Agree)**

1 2 3 4 5

13. I feel this organization is everyone's company.

****Rating Scale**: 1 (Strongly Disagree) to 5 (Strongly Agree)**

1 2 3 4 5

14. I feel a high degree of personal ownership in this organization.

****Rating Scale**: 1 (Strongly Disagree) to 5 (Strongly Agree)**

1 2 3 4 5

15. I feel this is my company.

****Rating Scale**: 1 (Strongly Disagree) to 5 (Strongly Agree)**

1 2 3 4 5

16. This is everyone's company.

****Rating Scale**: 1 (Strongly Disagree) to 5 (Strongly Agree)**

1 2 3 4 5

17. Most employees working for this organization feel they own the company.

****Rating Scale**: 1 (Strongly Disagree) to 5 (Strongly Agree)**

1 2 3 4 5

18. I find it difficult to consider this company as my own.

****Rating Scale**: 1 (Strongly Disagree) to 5 (Strongly Agree)**

1 2 3 4 5

### For Management (e.g., HR Manager):

19. Employees have clear career development paths within the organization.

Rating Scale: 1 (Strongly Disagree) to 5 (Strongly Agree)**

1 2 3 4 5

20. There is little career advancement for employees in this company.

****Rating Scale**: 1 (Strongly Disagree) to 5 (Strongly Agree)**

1 2 3 4 5

21. Supervisors in the organization are aware of employees' career development expectations.

****Rating Scale**: 1 (Strongly Disagree) to 5 (Strongly Agree)**

1 2 3 4 5

22. There are multiple suitable positions available for employees when they are promoted.

****Rating Scale**: 1 (Strongly Disagree) to 5 (Strongly Agree)**

1 2 3 4 5

23. Overall, the organization provides comprehensive training for employees.

****Rating Scale**: 1 (Strongly Disagree) to 5 (Strongly Agree)**

1 2 3 4 5

24. The organization arranges training for employees at regular intervals.

****Rating Scale**: 1 (Strongly Disagree) to 5 (Strongly Agree)**

1 2 3 4 5

25. The organization has formal training programs to teach new employees the skills needed for their jobs.

****Rating Scale**: 1 (Strongly Disagree) to 5 (Strongly Agree)**

1 2 3 4 5

26. The organization provides formal training to help employees advance in their careers.

****Rating Scale**: 1 (Strongly Disagree) to 5 (Strongly Agree)**

1 2 3 4 5

27. In the organization, performance is usually measured using objective and quantifiable results.

****Rating Scale**: 1 (Strongly Disagree) to 5 (Strongly Agree)**

1 2 3 4 5

28. Employee performance evaluations are based on objective and quantifiable results.

****Rating Scale**: 1 (Strongly Disagree) to 5 (Strongly Agree)**

1 2 3 4 5

29. Employees can propose reasonable suggestions to improve work methods.

****Rating Scale**: 1 (Strongly Disagree) to 5 (Strongly Agree)**

1 2 3 4 5

30. Managers are able to communicate openly and honestly with employees regularly.

****Rating Scale**: 1 (Strongly Disagree) to 5 (Strongly Agree)**

1 2 3 4 5

31. Managers often take into account the opinions of employees in the decision-making process.

****Rating Scale**: 1 (Strongly Disagree) to 5 (Strongly Agree)**

1 2 3 4 5

32. Employees in the organization can often decide their own work methods in many situations.

****Rating Scale**: 1 (Strongly Disagree) to 5 (Strongly Agree)**

1 2 3 4 5

33. The organization has a clear description of employees' job responsibilities.

Rating Scale: 1 (Strongly Disagree) to 5 (Strongly Agree)
1 2 3 4 5

34. Often, employees are also responsible for tasks outside their job responsibilities.

Rating Scale: 1 (Strongly Disagree) to 5 (Strongly Agree)
1 2 3 4 5

35. The job description for employees includes all the responsibilities they need to undertake.

Rating Scale: 1 (Strongly Disagree) to 5 (Strongly Agree)
1 2 3 4 5

36. The organization revises employees' job descriptions in a timely manner when necessary.

Rating Scale: 1 (Strongly Disagree) to 5 (Strongly Agree)
1 2 3 4 5

37. As long as employees are willing, they can stay in the organization.

Rating Scale: 1 (Strongly Disagree) to 5 (Strongly Agree)
1 2 3 4 5

38. It is very difficult for the organization to lay off employees.

Rating Scale: 1 (Strongly Disagree) to 5 (Strongly Agree)
1 2 3 4 5

39. The organization can ensure job stability for employees.

Rating Scale: 1 (Strongly Disagree) to 5 (Strongly Agree)
1 2 3 4 5

40. If the organization encounters economic difficulties, layoffs will be the last resort.

Rating Scale: 1 (Strongly Disagree) to 5 (Strongly Agree)
1 2 3 4 5

41. The organization will allocate bonuses to employees based on its profitability.

Rating Scale: 1 (Strongly Disagree) to 5 (Strongly Agree)
1 2 3 4 5
